# The failure mechanism of the Baishi landslide in Beichuan County, Sichuan, China

**DOI:** 10.1038/s41598-024-67402-1

**Published:** 2024-07-30

**Authors:** Ran Tang, Suichuan Ren, Juntao Li, Peng Feng, Huajin Li, Ren Deng, Daxin Li, Kiyonobu Kasama

**Affiliations:** 1 School of Architecture and Civil Engineering, Chengdu University, Chengdu, Sichuan China; 2 Key Laboratory of Hydraulic and Waterway Engineering of the Ministry of Education, Chongqing Jiaotong University, Chongqing, China; 3 Sichuan Engineering Research Center for Mechanical Properties and Engineering Technology of Unsaturated Soils (Chengdu University), Chengdu, Sichuan China; 4 Sichuan Institute of Geological Engineering Investigation Group Co.Itd, Chengdu, Sichuan China; 5 Department of Civil Engineering, Kyushu University, Fukuoka, Japan

**Keywords:** Natural hazards, Geology

## Abstract

The Baishi landslide was located in the western part of Beichuan County, Sichuan Province, China. The landslide experienced multiple minor collapses at the front part, accompanying with numerous tensile cracks. To comprehensively grasp the stability conditions and predict the moment of failure of the landslide, deformation monitoring of the landslide has been carried out from the moment that the landslide was reported until it failed. This study analyzed the different phases of landslide deformation and its failure mechanism through the analysis of monitoring data. The result showed that the failure manifests both the retrogressive and advancing features. The landslide was divided into several zones based on the spatial variation of the deformation characteristics. Moreover, the improved tangential angle criterion is applied to categorize the deformation phases of a landslide. Investigating the surface displacement vectors and vector angles of landslides plays a significant role for ascertaining the failure and sliding mechanism. The monitoring results revealed that the front part of the landslide played a key role in the stability of the landslide. Therefore, the monitoring data from this zone were crucial for predicting the moment of complete landslide failure.

## Introduction

**Figure 1:**
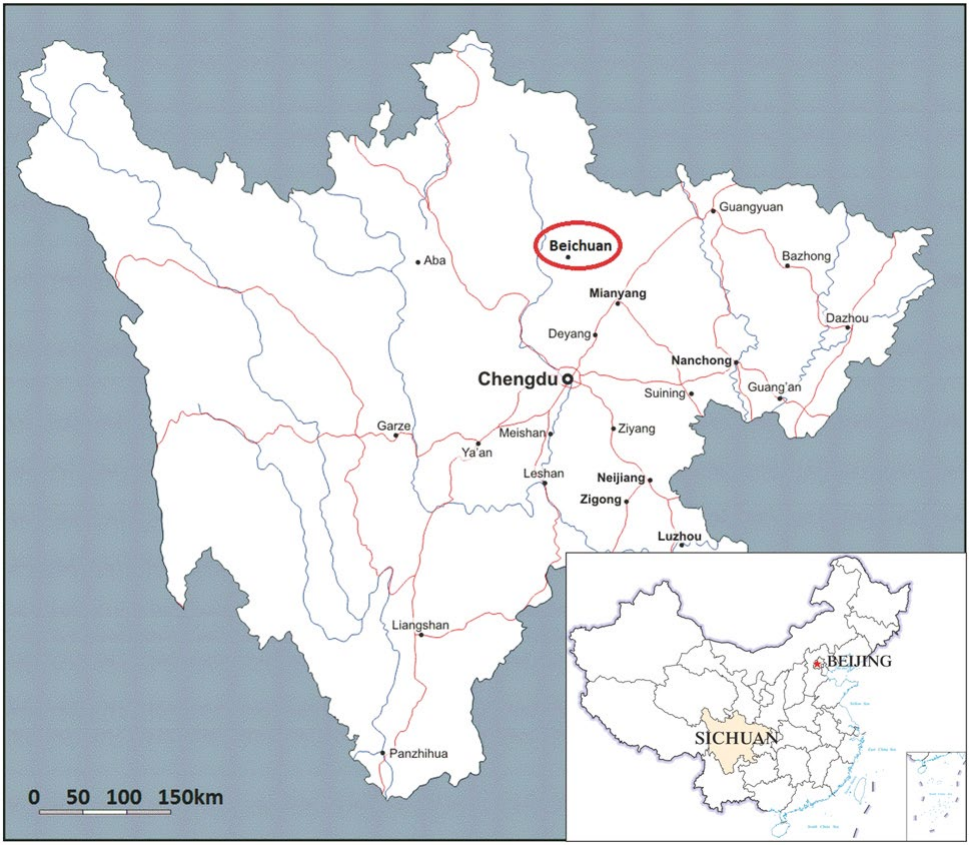
Location of the Baishi landslide in Beichuan County.

**Figure 2:**
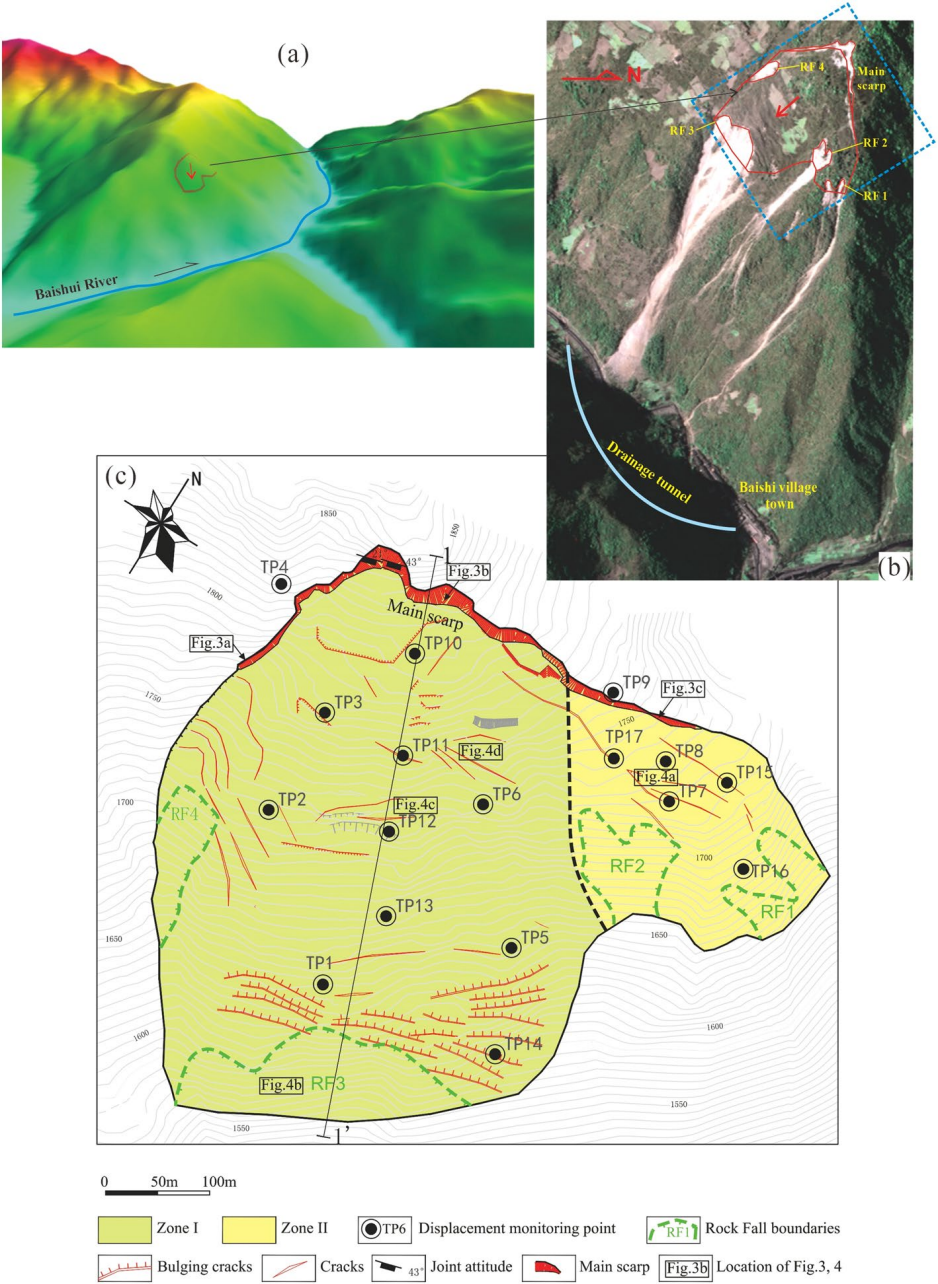
(**a**) Digital terrain model of Baishi landslide; (**b**) remote sensing imagery (Sichuan Provincial Remote Sensing Center, January 29, 2007); (**c**) displacement monitoring points layout and surface deformation features in January 2007.

**Figure 3:**
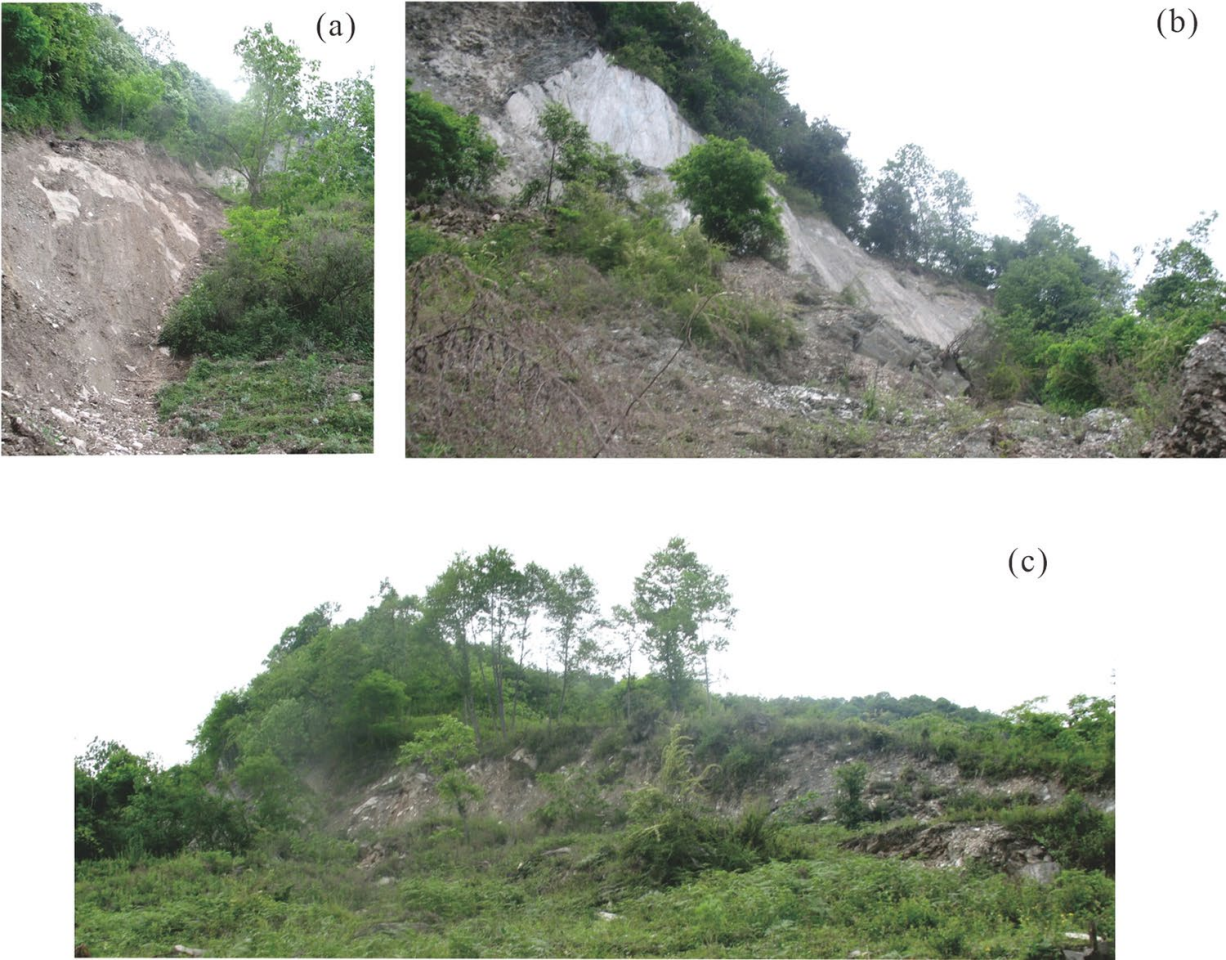
Main scarp of Baishi landslide: (**a**) western boundary; (**b**) central point of zone I; (**c**) eastern slide boundary in zone II.

**Figure 4:**
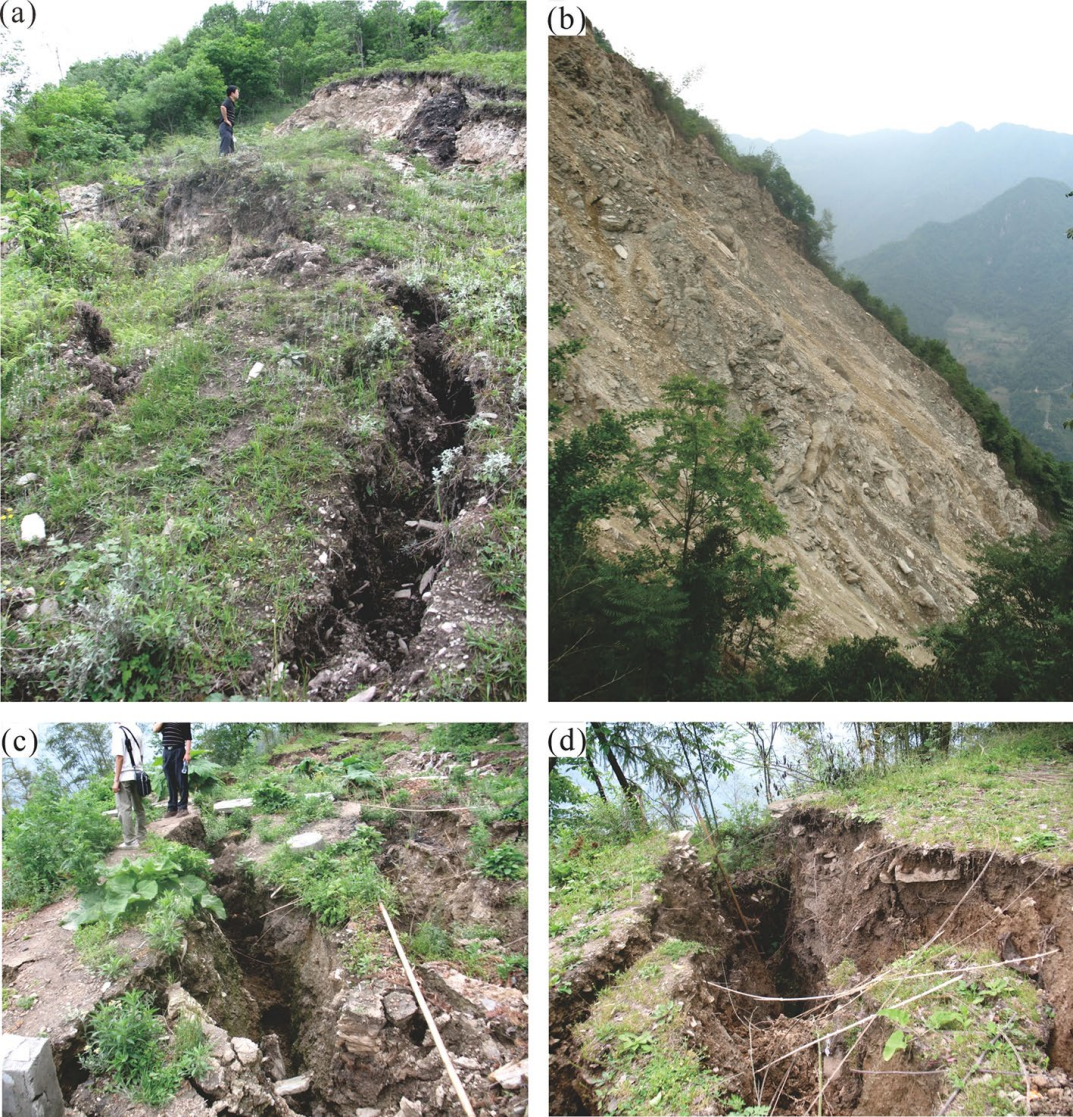
Deformation features of Baishi landslide: (**a**) Tension cracks in zone II; (**b**) RF3 collapse; (**c**) Cracks behind monitoring point TP12; (**d**) Cracks near monitoring point TP11.

The Baishi landslide is located in the western part of Beichuan County, Mianyang, Sichuan Province (Fig. 1). The landslide became apparent due to multiple minor collapses and falls at the front of the landslide as well as numerous tensile cracks in the slope in December 2006 (Figs. 2 and 3). The part of the slope that was affected was approximately 300 m long, 260 m wide, and 25 m thick on average, and about 2 × 10^6^ m^3^ in total volume (Figs. 2a and b and Fig. 4). If the overall landslide had collapsed, a barrier lake would have formed in the Baishui River, and more than 1400 people would be endangered. To give an accurate forecast of the deformation process of the Baishi landslide, a comprehensive deformation monitoring program has been implemented on the landslide. Monitoring started in January 2007 and was continued until the landslide failed at the end of July 2007.

The deformation of slopes can be elucidated through three stages of creep behavior^1,2^. The classical creep curve delineates the three stages, forming the basis for the majority of time-of-failure predictions^3^. Geotechnical engineering frequently faces slope stability challenges, and early warning systems can reduce risks in a vast range of fields, including landslides^4^. The accuracy of time-of-failure predictions primarily relies on the chosen parameters and methods. The parameters of the slope are ascertained through a combination of monitoring and testing processes. Various prediction methods influence the outcomes of landslide assessments. Fukuzono^5^ proposed the inverse velocity method, an empirical formula. The formula, derived from large-scale laboratory tests simulating rain-induced landslides in soil, embodies a time-dependent failure relationship. Empirical parameters within the formula are linked to the occurrence conditions of landslides. The empirical parameter values proposed by Fukuzono demonstrate greater effectiveness in predicting landslides influenced by rainfall. It is crucial to optimize empirical parameters and methods according to specific landslide situations to align with real-world conditions^6–9^. Further, the semi-empirical methods were proposed through a mathematical generalization of the solution proposed by Fukuzono^10–12^. The empirical methods exhibit the unstable prediction accuracy, lacking theoretical support. The semi-empirical methods have only been applied to predict rock-specimen tests and volcanic eruptions. The methods for defining warning thresholds can provide reliable predictions according to different phenomena. Because methods are based on the physical behavior of the environment.

Nowadays, scholars have proposed many threshold methods according to different phenomena^13–15^. For example, rainfall-induced slope failure is a common form of landslide, so determining rainfall thresholds of landslide can predict landslide failure^16–19^. Prediction methods rely solely on a single landslide-triggering factor as the threshold frequently overlook other potential triggering factors. Accurate judgment of the deformation phase of a slope is pivotal for early warning, and the comprehensive analysis of temporal-spatial deformation evolution is crucial for precise slope prediction. Displacement is considered as the most straightforward indicators to recognize instability and to characterize different deformation stages^20,21^, and the displacement can be categorized into two groups including deep displacement and surface displacement. The deep displacement monitoring method has been extensively employed to track deformation characteristics of a sliding body^22,23^. However, this method cannot be easily employed for full landslide phase division since that the inclinometer can be sheared off during the process of landslide. Surface monitoring data are widely used for landslide phases division due to the fact that surface monitoring can reveal the full phases of landslide movement^24–26^. Xu et al.^27^ extensively analyzed the characteristics of displacement curves, velocity curves, and acceleration curves in numerous landslide cases. In the literature, they proposed a new method to describe the tangential angle of the displacement–time curve, and a new criterion based on the angle was used to divide the tertiary creep (accelerated) phase into three sub-phases: initial acceleration, medium acceleration, and the critical failure. Moreover, applying the prediction method that establishes parameter thresholds according to the deformation mechanism and characteristics of landslides proves more effective across various practical engineering scenarios. Researchers discussed a method for forecasting the time of failure by establishing an alert acceleration threshold value, which has been applied to investigate numerous landslides^8,28–30^. Adequate monitoring data were obtained through monitoring during the movement of the Baish landslide. Scholars have studied the Baishi landslide, including analyzing the deformation mode and predicting the sliding time by analyzing the surface monitoring data of the Baishi landslide^31,32^, and analyzing the dynamic evolution characteristics of the landslide by studying the remote sensing data^33^.

The objective of this study was to find out if a careful analysis of the surface displacement data could lead to a definition of the development of the landslide deformation phases and failure mechanism and a realistic early warning of the moment of failure.

## Characteristics of the Baishi landslide

Manifest evidence of deformation was first observed in December 2006, after which the deformation features developed rapidly. In January 2007, the main scarp had extended to more than 390m, with a vertical displacement between 2 and 30m (Figs. 2c and 3). Four collapses occurred along the boundary of the landslide. RF1 and RF2 (in green in Fig. 2c) were located in zone ii. RF3, which occurred first, was located at the front of zone I, numerous bulging cracks developed behind it, and its size expanded with increasing rock fall (Figs. 2c and 4b). RF4 was developed towards a gully beyond the western boundary of zone I, while numerous tension cracks developed behind it (Fig. 2c). The area between monitoring points TP10 and TP12 was another most severely deformed region. The ground surface there was torn into pieces by cracks (Figs. 2c, 4c, and d).

## Monitoring methodology

**Figure 5:**
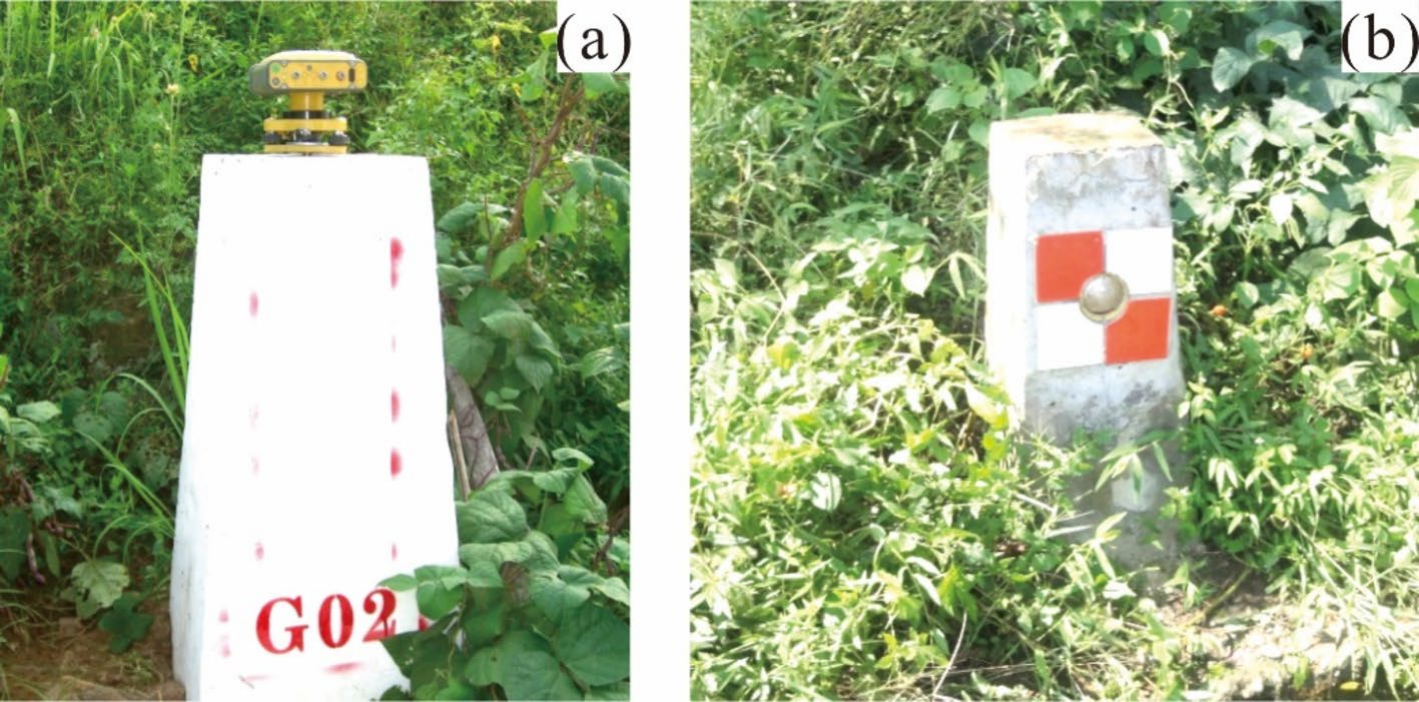
(**a**) Forced centering pile in monitoring station; (**b**) monitoring point on the landslide.

Certain related literature suggests that field monitoring plays a pivotal role in analyzing the failure mechanism of large-scale landslides^34–38^. Monitoring items include the following: surface horizontal and vertical displacements, rainfall, and relative displacements of cracks. The horizontal and vertical displacements were measured by a robotic total station, whose angle measurement accuracy was 0.5″ and the range measurement accuracy was 1mm + 1ppm. The monitoring stations were fixed in two stable locations on the opposing hill where the altitude was close to the landslide and the horizontal distance to the landslide was about 900 m. Forced centering reinforced concrete piles were used for the robotic total station with a height of 1.2 m above the ground and 0.8 m below the ground (Fig. 5a). The monitoring points on the landslide were made by small square concrete piles with a triangle prism fixed in, making a crosshair across the center of the prism (Fig. 5b). The datum network was calibrated by GPS once in three months. After the first field survey and mapping, 17 surface displacement monitoring points were installed on the landslide successively (Fig. 2c).

## Analysis of the deformation monitoring data and deformation features

### Division of the landslide into subareas

The Baishi landslide was divided into two subareas, Zone I and Zone II (Fig. 2c) for the following three reasons, (1) Zone I showed more intense macroscopic deformation features than Zone II. (2) The average surface slope angle of Zone I (more than 40°) was much larger than that of Zone II (around 27°). (3) There were major differences in moving direction and moving velocity between Zone I and Zone II. The overall displacement direction in Zone I was 160° to 170°, in Zone II 140° to150°; the average velocity in Zone I was around 78 mm/day, and in Zone II was around 20 mm/day. Zone I was the main body of the Baishi landslide, so the monitoring data of Zone I are the main objects of our analysis.

### Division into different stages of deformation

A large number of landslide monitoring data shows that a creep slope always goes through three phases from the beginning to the final failure. Namely, primary creep (“decelerated”), secondary creep (“steady-state”), and tertiary creep (“accelerated”)^2^. Xu et al.^27^ based on a statistical analysis of a considerable number of landslide cases, proposed criteria named “improved tangential angle criterion” to divide the three deformation phases depending on a displacement–time curve, furthermore, the criteria was used to divide accelerated phase into three sub-phases. That is, when the improved tangential angle > 45°, the slope deformation enters the initial deformation sub-phase (primary stage); when the improved tangential angle > 80°, the slope deformation enters the medium accelerated deformation sub-phase (intermediate stage); when the improved tangential angle > 85°, the slope deformation enters the highly accelerated (the critical slide) phase.

The definition made as follows:

1 $$T(i) = \frac{s(i)}{v}$$

Here, *s*(*i*) is the cumulative displacement of time *t*_*i*_; *v* is the average velocity of the secondary creep phase; *T*(*i*) is the relative time corresponding to monitoring time *t*_*i*_, with the same dimension and unit as time.

An improved expression of the tangential angle αi can be obtained^27^:

2 $$\alpha_{i} = \arctan \frac{T(i) - T(i - 1)}{{t_{i} - t_{i - 1} }} = \arctan \frac{\Delta T}{{\Delta t}}$$

where *α*_*i*_ is the tangential angle of the *T*-*t* curve; *t*_*i*_ is the *i*th of monitoring time; Δ*t* is the monitoring period, such as 1 day, 1 week, etc.; Δ*T* represents the change in volume per unit time.

**Figure 6:**
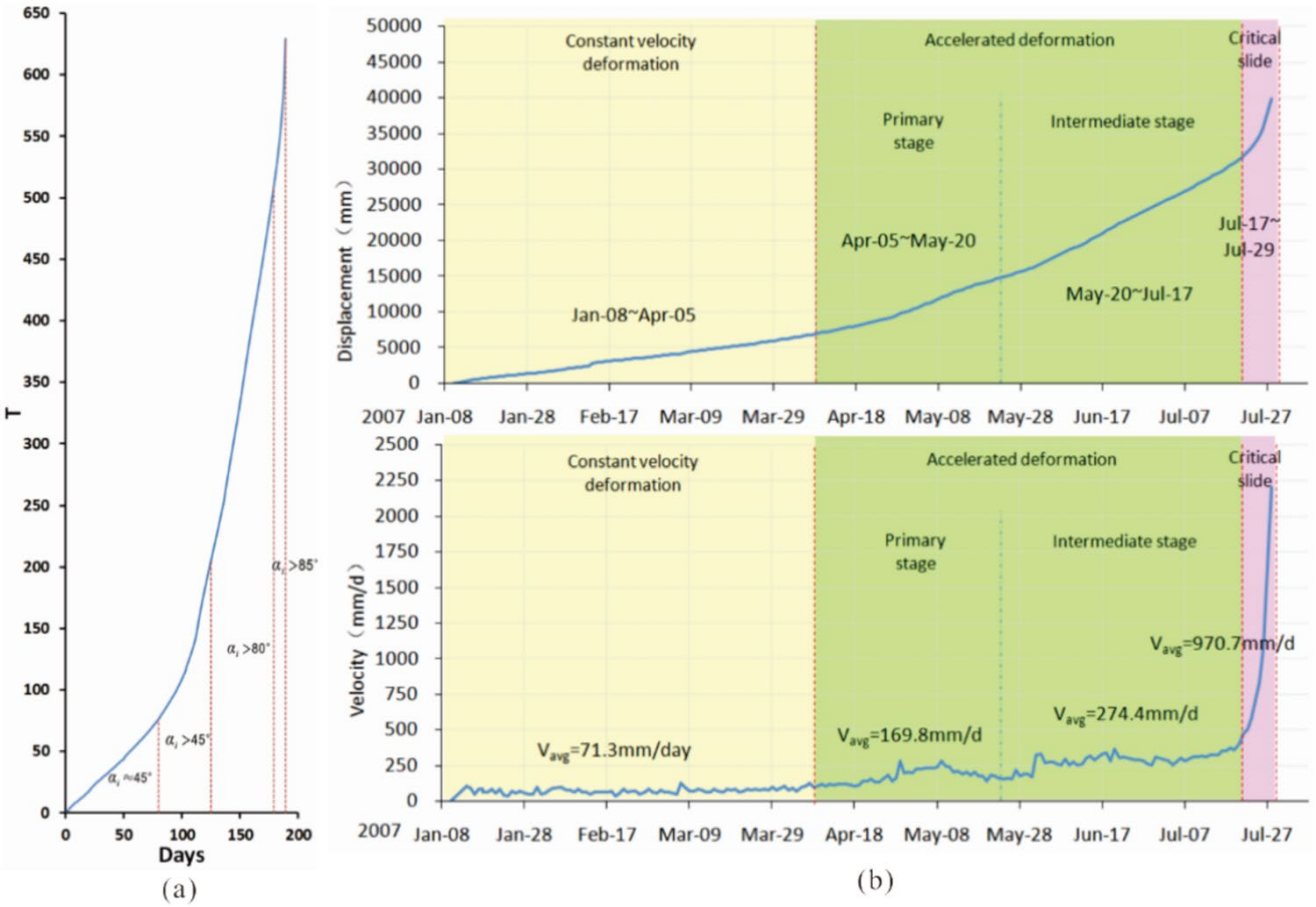
(**a**) *T-t* curve of Baishi landslide (reproduced after Xu et al.^27^); (**b**) Deformation phases of Bashi landslide based on the displacement and velocity curves from TP1.

Traditional field investigation of landslide deformation and the evaluation based on the monitoring data of landslides were used for landslide phases division. However, the results based on the traditional method highly depend on the researcher^39^. As a quantitative method, the improved tangential angle criterion is employed for analyzing landslide phases division, which can acquire the accurate determination of the deformation phases of landslides based on the data obtained from monitoring^40^. Based on the improved tangential angle criterion (Fig. 6a), and in combination with the surface deformation features observed, the deformation process was divided into three phases, 1: a constant velocity deformation (“steady-state”) phase (from January 8 to April 5), 2: an accelerated deformation phase (from April 5 to July 17), 3: the critical slide phase (from July 17 to July 29), where the second phase can be divided into two sub-phases, a primary stage(April 5 to May 20) and an intermediate stage (May 20 to July 17) (Fig. 6b).

Numerous landslide monitoring processes show that the accelerated deformation phase is leading to the failure of the landslide. Therefore, this phase plays a major role in the forecasting of the moment of occurrence of a landslide.

### Constant velocity deformation phase

**Figure 7:**
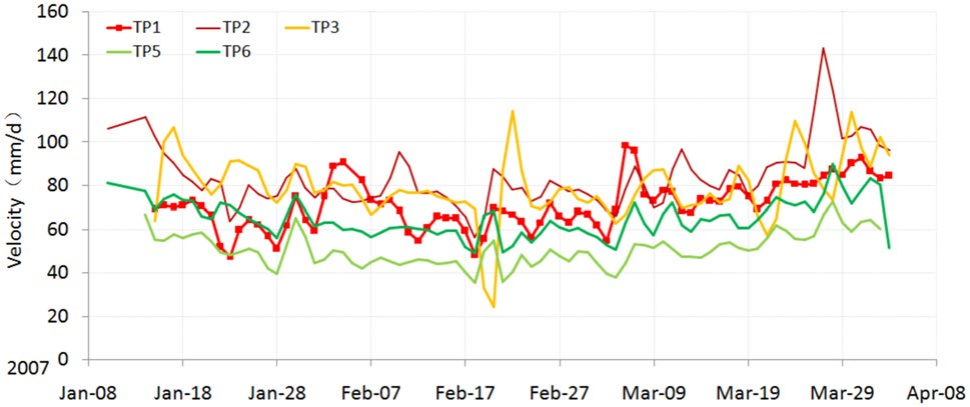
Velocity curves during the constant velocity deformation phase.

At the beginning of monitoring, we assumed that the Baishi landslide had already entered into the primary accelerated deformation phase because the velocity of the landslide had reached 40mm/d to 100mm/d in the first week, which was higher than most landslides. Meanwhile, the terrain surface deformation features were severe. However, after analyzing the displacement and velocity curves, we concluded that the landslide was still in the constant velocity deformation phase^41^, for the velocity did not continuously increase during the first two months (Fig. 7).

During the constant velocity deformation phase, the landslide showed a characteristic way of advancing: the velocities of the monitoring points in the middle and upper part of the landslide (TP2, TP3, TP10, TP11, TP12, TP13, TP6) are higher than in the toe area (TP1, TP5, TP14) (Table. 1). The front part heaved up, and many bulging cracks occurred in the toe area near the RF3 collapse (Fig. 2c). This means that the frontal part of the landslide was pushed by the middle and upper part of the landslide.

**Table 1:** Velocities of the monitoring points during each deformation phase.

Monitoring point	Constant phase	Accelerated phase (primary)	Accelerated phase (intermediate)	Critical slide phase
TP1	71 mm/d	170 mm/d	274 mm/d	971 mm/d
TP2	84 mm/d	188 mm/d	296 mm/d	1402 mm/d
TP3	79 mm/d	183 mm/d	236 mm/d	2004 mm/d
TP5	51 mm/d	123 mm/d	168 mm/d	386 mm/d
TP6	65 mm/d	148 mm/d	213 mm/d	483 mm/d
TP10	91 mm/d	176 mm/d	251 mm/d	1176 mm/d
TP11	83 mm/d	168 mm/d	261 mm/d	1364 mm/d
TP12	90 mm/d	190 mm/d	348 mm/d	destroyed
TP13	94 mm/d	186 mm/d	291 mm/d	729 mm/d
TP14	52 mm/d	118 mm/d	192 mm/d	591 mm/d

The entry of the accelerated deformation phase is the premise of slope failure^6,27^. That means, at the previous two deformation phases, no matter how high the velocity was or how severe the microscopic deformation features were, for a creeping landslide, a sudden, extremely rapid failure will not happen normally. According to the analysis above, the landslide was still in secondary creep (“steady-state”), so the overall landslide will not collapse in the short term. The Baish landslide mass might induce the formation of a barrier lake within the Baishui River, potentially culminating in exacerbated consequences. Consequently, there arises a necessity to excavate a drainage tunnel on the mountain opposite the slope to mitigate disaster risks through drainage. The drainage tunnel started construction on March 14, 2007, completed on June 16, 2007, with a length of 678 m (Fig. 2b).

### Primary accelerated deformation stage

**Figure 8:**
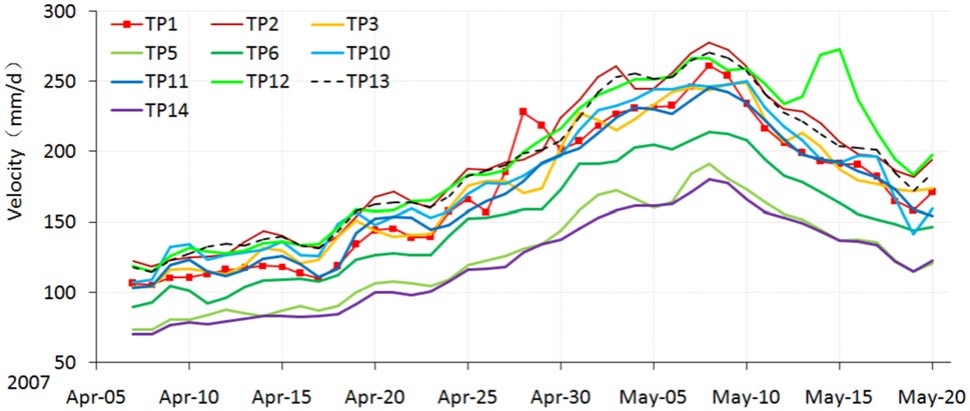
Velocity curves during the primary stage of the accelerated deformation phase.

Around April 5, 2007, the landslide entered into the primary stage of the accelerated deformation phase. Compared with the constant velocity deformation phase, a distinct change occurred. The velocity curves show the rising shape (Fig. 8), and the averaged velocity increasing sharply (Table 1).

**Figure 9:**
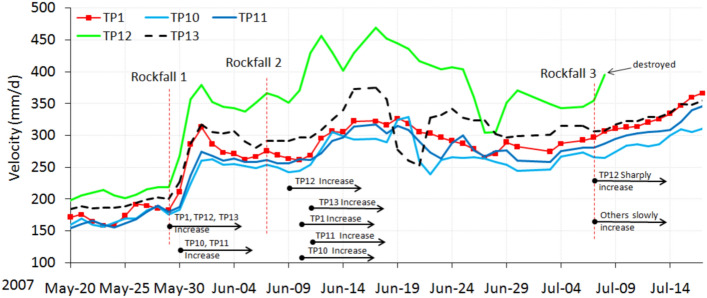
Velocity curves during the intermediate stage of the accelerated deformation phase.

**Figure 10:**
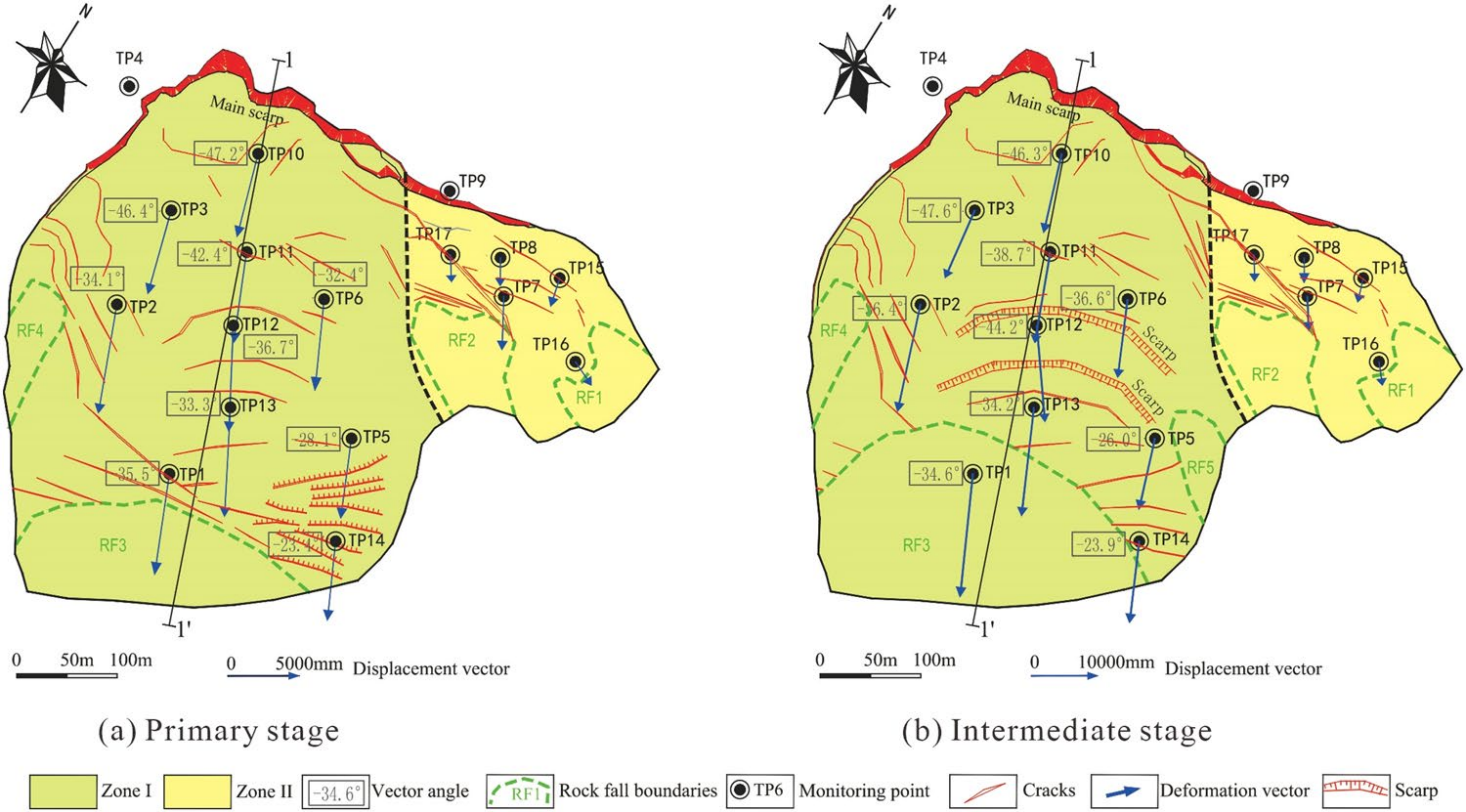
Displacement vectors and cracks during the accelerated deformation phase: (**a**) primary stage; (**b**) intermediate stage.

The RF3 collapse area became larger, and the cracks near the top of RF3 and RF4 extended and became connected (Fig. 9). The cracks between the monitoring points TP11 and TP12 (Fig. 10a) further developed, some of them up to several meters deep.

### Intermediate stage of the accelerated deformation phase

Around May 20, 2007, the landslide entered into the intermediate stage of the accelerated deformation phase. The RF3 collapses occurred every day, and its area expanded rapidly; cracks near the RF3 and RF4 zones connected; the RF5 collapse appeared in the toe area near the TP5 monitoring point, and the deep cracks between TP11 and TP12 became scarps. During this phase, the landslide started to show some characteristics of retrogressive mode. (Fig. 10b).

The velocity curves of the monitoring points along Sects. 1–1’ were rising and falling greatly (Fig. 9), caused by three large mass rock fall events in the toe area, which happened on May 29, June 7, and July 7. Each large mass rock fall that occurred in RF3 had an unloading effect on the toe area, after which the velocity of the landslide rose sharply (Fig. 9). The response of the deformation velocity to the decrease of load became more and more violent, which showed that the landslide was reaching a critical phase. Different parts of the landslide responded differently. The velocity of TP1, TP12 and TP13, which are situated in the middle and toe area, increased severely on the day the rock fall 1 event happened, but the velocities of TP10 and TP11, which are situated near the back scarp of the landslide, increased only the next day (Fig. 9). After the rock fall 2 event, the velocity of TP12 increased on the third day, the other monitoring points responded one or two days later. After the rock fall 3 event, the curve of TP12 rose sharply but then was destroyed because of excessive deformation. Retrogressive deformation features are evident due to unsynchronized deformation velocities. The curves of the other monitoring points rose only slowly (Fig. 9). These deformation characteristics show that the slide body below TP12 was separated from the part above TP12. So the toe area controlled the failure of the landslide. It all depended on how much the resisting force due to the toe area was able to resist the land sliding. The monitoring data of point TP1 in the toe area played a significant role in the possibility of early warning for the start of the sliding phase.

### Critical sliding phase

After July 7, 2007, the volume of rock fall mass was increasing considerably in the toe area, until July 26 the volume was about 200m^3^/d. The velocity of the displacement of every monitoring point was continuously increasing (Fig. 13a), to much higher values than in the previous stage. On July 17, the velocity of the TP1 monitoring point became larger than that of other points (Fig. 13a) which proves that the toe area had almost lost its resisting force capacity. This can be regarded as the signal of entering the critical sliding phase.

**Figure 11:**
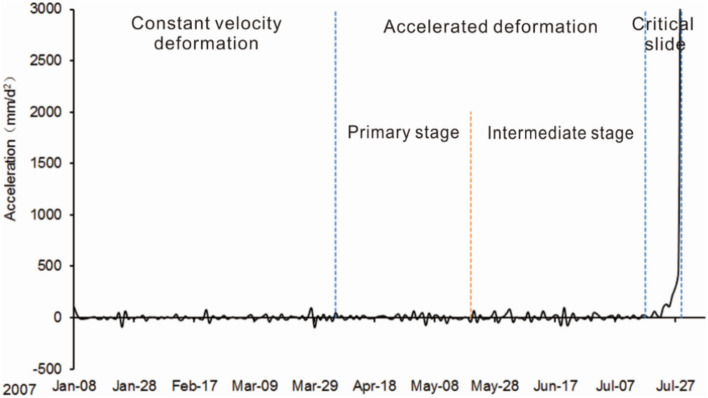
Acceleration curve of monitoring point TP2.

**Figure 12:**
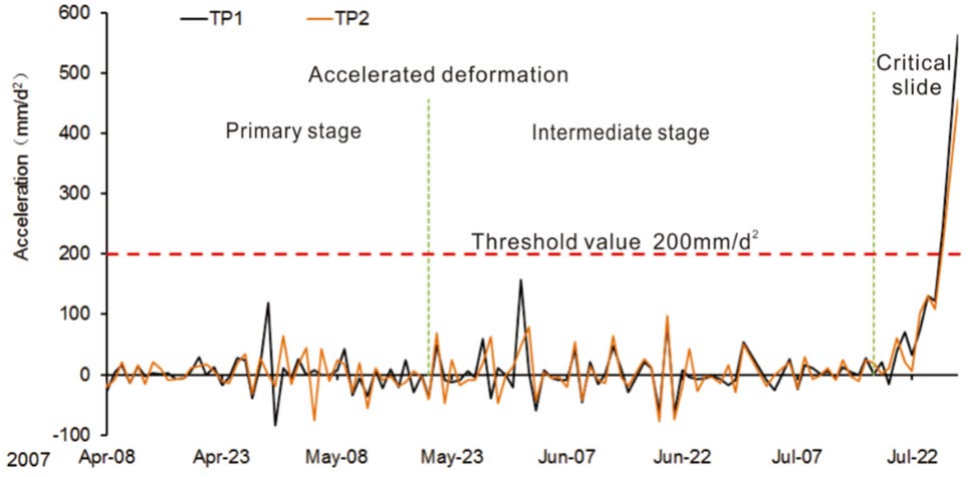
Acceleration curve of monitoring point TP1 and TP2 after the entry of accelerated deformation phase, before Jul 28, 2007.

The acceleration-time curve for the Baishi landslide can be found in Fig. 11, the figure shows that before the landslide entered into a critical sliding phase, the acceleration values kept fluctuating around 0; even if it was in an accelerated deformation phase. Compared with displacement and velocity curves (Fig. 6b), which increased gradually during the accelerated deformation phase. Figure 11 also indicates that a sharp rise occurs right after the entry of the critical sliding phase. The acceleration shows completely different characteristics before and after the slope enters the critical failure phase, showing significant catastrophic characteristics at the entry of the critical failure phase^27^. The acceleration-time curve of TP1 and TP2 after the entry of the accelerated deformation phase can be found in Fig. 12, the figure shows the acceleration varying amplitude was confined in a certain range, which can be regarded as the threshold value, around 160mm/d^2^. After considering engineering experience and response time to disasters, the given value of the pre-warning criterion that led to the overall failure was 200mm/d^2^, which is a little larger than 160 mm/d^2^.

**Figure 13:**
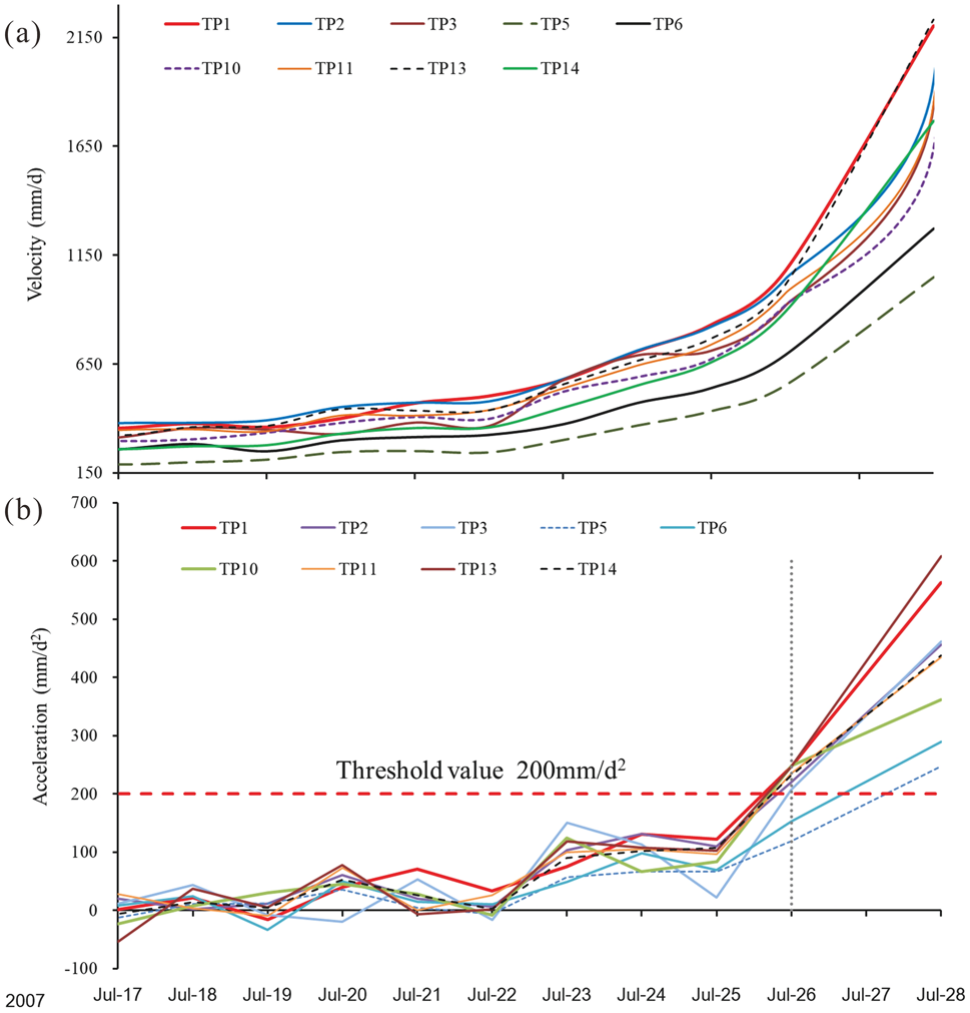
(**a**) Velocity curves during the critical sliding phase, (**b**) acceleration curves during the critical sliding phase.

**Figure 14:**
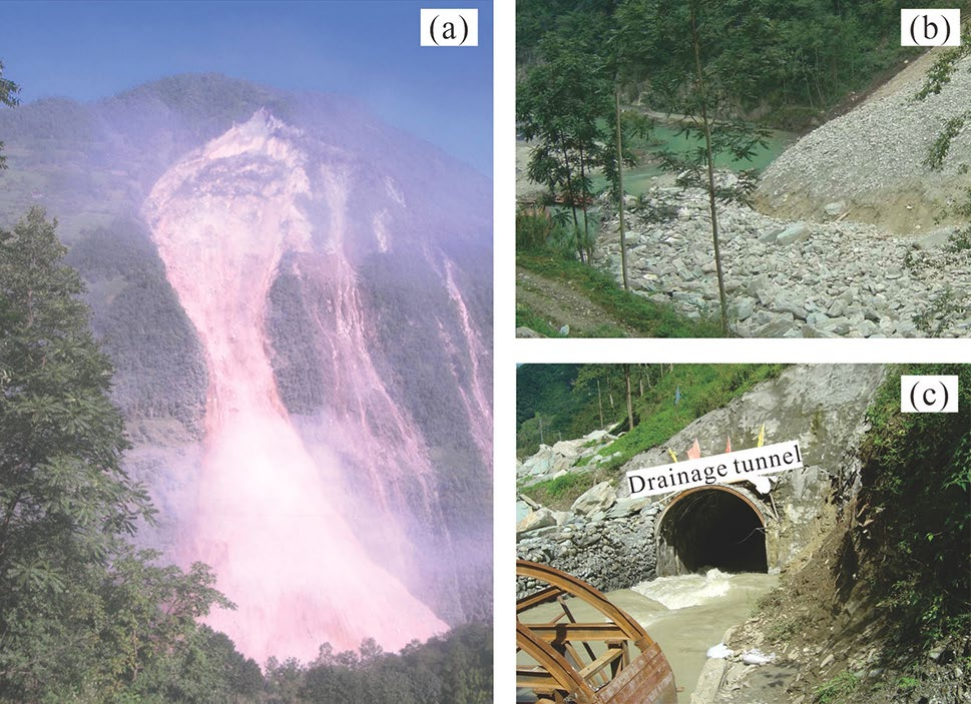
(**a**) Full view of Baishi landslide when sliding on July 30, 2007; (**b**) landslide dam and barrier lake; (**c**) drainage tunnel releasing the water of barrier lake.

On July 26, the acceleration value of most monitoring points became larger than the pre-set warning threshold of 200mm/d^2^, except TP5 and TP6 (Fig. 13b). On that day the overall failure warning was issued. On July 28, 2007, the overall landslide started to collapse, till July 30, 2007, the sliding mass blocked the Baishui River to form a barrier lake (Figs. 14a and b). The water in the barrier lake was successfully drained downstream by the tunnel (Fig. 14c); the slope failure did not cause life and property losses to the local and upstream residents.

## Formation mechanism and failure mechanism of the landslide

### Formation mechanism of the landslide

**Figure 15:**
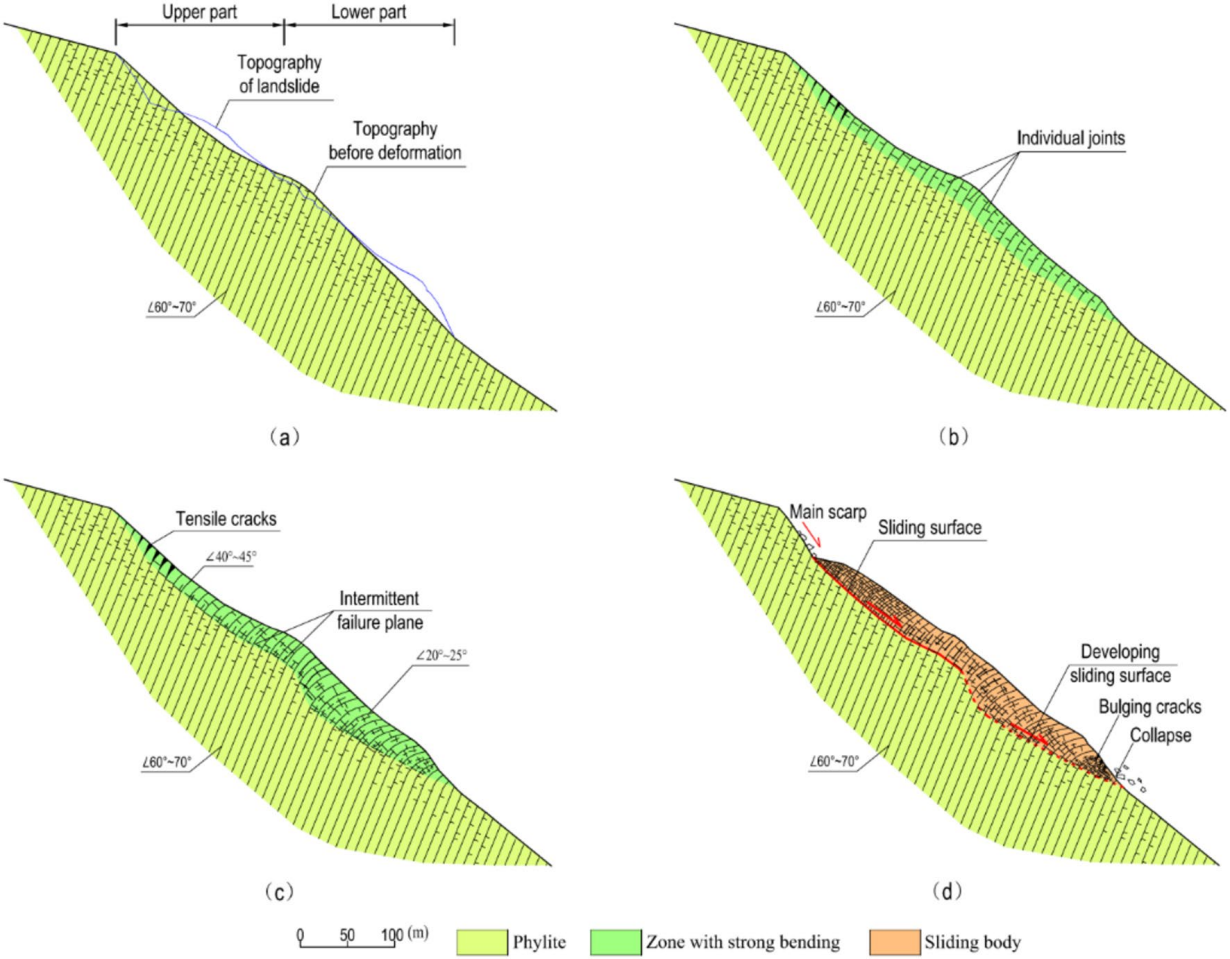
Development of the Baishi landslide: (**a**) topography before bending deformation started; (**b**) bending deformation of strata near the terrain surface; (**c**) bending deformation progressing deeper inside; (**d**) sliding surface formed from the top-scarp downwards.

**Figure 16:**
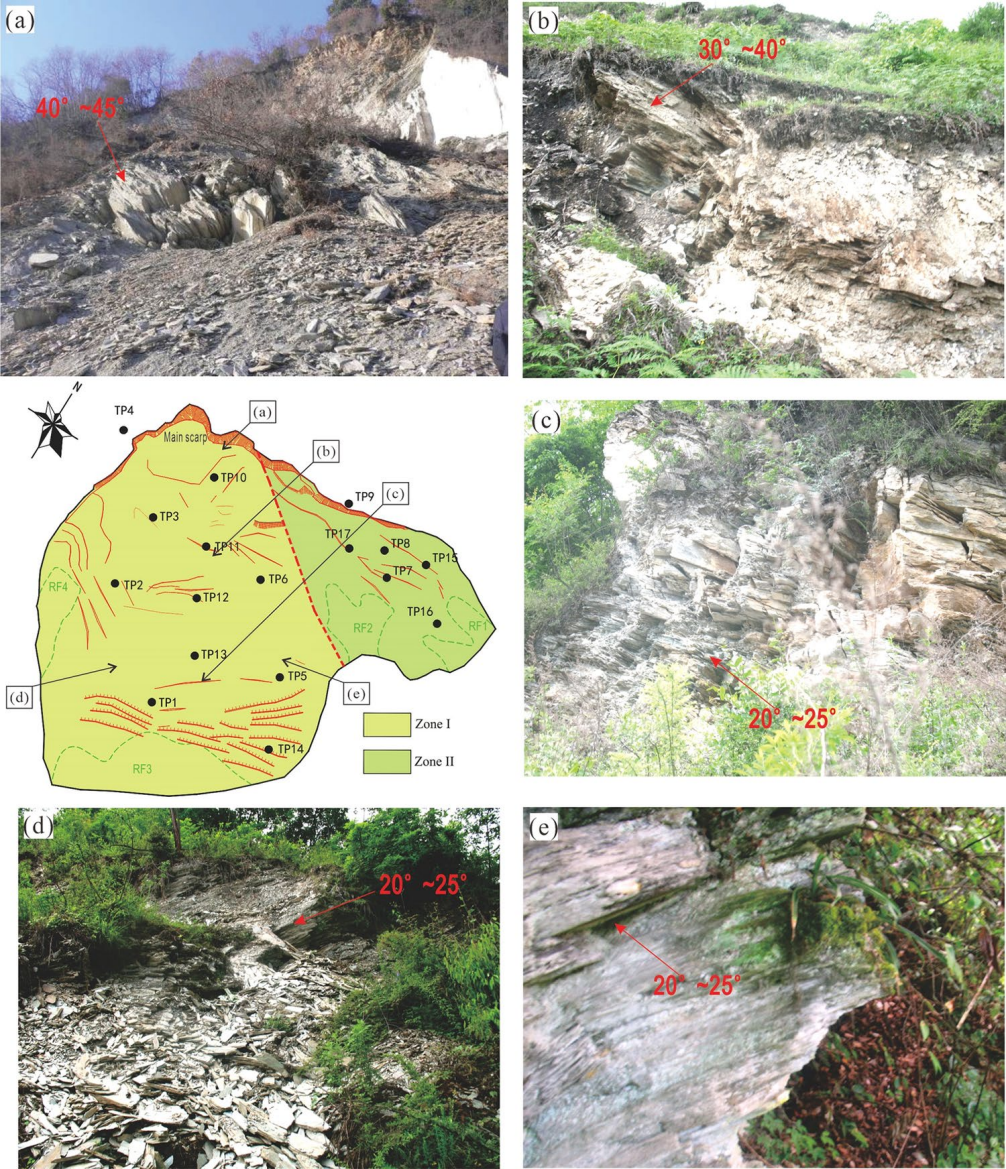
Bending deformation phenomena: (**a**) highly weathered phyllite near main scarp with a dip angle of 40° ~ 45°; (**b**) weathered phyllite near TP11 with a dip angle of 30°–40°, (**c**) Weathered phyllite outcrop in the lower part with a dip angle 20°–25°; (**d**) highly weathered phyllite outcrop with a dip angle 20° ~ 25°; (e) weathered phyllite outcrop in the lower part with a dip angle 20°–25°.

Our field investigations showed that the landslide was developed in highly weathered Maoxian group phyllite rock, on a typical anti-dip slope (Figs. 15 and 16). Slopes with this kind of structure are prone to deform in the modes of tensile bending and toppling^42^.

Based on former topographic data, we inferred the initial topography of Sect. 1–1’ before the bending deformation started (Fig. 15a).

First bending deformation occurred in a shallow near surface zone due to gravity, and the toe area started to uplift (Fig. 15b). Then, the bending deformation extended from the surface zone to deeper inside the slope (Fig. 15c). The initial topography can be divided into two parts (Fig. 15a), the average slope angle of the upper part was about 35°, and the average slope angle of the lower part was about 45°. The shape of the upper part of the slope was slightly concave on section, the lower part almost has no fluctuation. The shape and slope angle of the upper part slope go against to bending deformation developing, instead, for the lower part, the bending deformation was easier to go deeper comparatively. The variation in bending deformation depth between the upper part and the lower part, influencing the geometry of the sliding surface, were shown through the dip angle of the highly weathered rock on the surface of the landslide. The dip angle of bedrock ranges from 60°—70°, in the upper part, at the foot of the main scarp, the dip angle varies from 40° to 45° (Fig. 16a), in the deep fissures near TP11, the dip angle varies from 30° to 40° (Fig. 16b), and in the lower part, several outcrops show the dip angle of weathered rock varied from 20° to 25° (Figs. 16c, d, and e),

As the deformation increased, the lower part of the slope gave the upper part more deformation space, while tensile cracks developed at the top of the upper part, and an intermittent failure plane, composed of individual joint planes oriented parallel to the slope surface, was generated, which separated the zone of strongest bending deformation from the less disturbed inner rock mass. This intermittent plane developed afterward into the sliding surface. The first severe deformation occurred at the top of the slope in the summer of 2003, based on the description of the local villagers, the main scarp was formed suddenly with a height of several meters, and then the height increased and extended every year. In the summer of 2006, the bulging cracks appeared in the toe area, after that, RF3 started to collapse, and at the end of 2006, RF1, RF2, and RF4 started to collapse. Consequently, the sliding surface was developed from the upper part of the slope to the lower part of the slope (Fig. 16d).

### Failure mechanism of the landslide

#### Surface displacement vectors and vector angles

The displacement vector angle can reflect the dynamic condition and the sliding mechanism of a landslide, which is a very useful parameter for failure mechanism analysis. The displacement vector angle is defined as the *arctan* (accumulative vertical displacement/horizontal accumulative displacement).

Suppose *x*_*i*_ are the horizontal displacement measured each time and* y*_*i*_ are the vertical displacement measured each time; *X*_*i*_ and *Y*_*i*_ are the accumulative horizontal and the accumulative vertical displacement since monitoring started to time (*t*_*i*_). The *X*_*i*_, *Y*_*i*_, *X*_*i*+*1,*_ and *Y*_*i*+*1*_ can be calculated as follows:

3 $$\begin{gathered} X_{i} = \sum\limits_{n = 1}^{i} {x_{n} } = x_{1} + x_{2} + x_{3} + \cdots { + }x_{i} ,\;X_{i + 1} = \sum\limits_{n = 1}^{i + 1} {x_{n} } \hfill \\ Y_{i} = \sum\limits_{n = 1}^{i} {y_{n} } = y_{1} + y_{2} + y_{3} + \cdots { + }y_{i} ,\;Y_{i + 1} = \sum\limits_{n = 1}^{i + 1} {y_{n} } \hfill \\ \end{gathered}$$

$\mathop{v}\limits^{\rightharpoonup} _{{i{ + }1}}$ is the surface displacement vector during time (*t*_*i*_) to time (*t*_*i*+*1*_); $\mathop{V}\limits^{\rightharpoonup} _{i}$ and $\mathop{V}\limits^{\rightharpoonup} _{{i{ + }1}}$ are the accumulative surface displacement vectors at time (*t*_*i*_) and time (*t*_*i*+*1*_). The $\mathop{V}\limits^{\rightharpoonup} _{i}$ and $\mathop{V}\limits^{\rightharpoonup} _{{i{ + }1}}$ can be calculated as follows:

4 $$\begin{gathered} \mathop{V}\limits^{\rightharpoonup} _{i} = \sum\limits_{n = 1}^{i} {\mathop{v}\limits^{\rightharpoonup} _{n} } = \mathop{v}\limits^{\rightharpoonup} _{1} + \mathop{v}\limits^{\rightharpoonup} _{2} + \mathop{v}\limits^{\rightharpoonup} _{3} + \cdots { + }\mathop{v}\limits^{\rightharpoonup} _{i} \hfill \\ \mathop{V}\limits^{\rightharpoonup} _{i + 1} = \mathop{V}\limits^{\rightharpoonup} _{i} + \mathop{v}\limits^{\rightharpoonup} _{i + 1} = \sum\limits_{n = 1}^{i} {\mathop{v}\limits^{\rightharpoonup} _{n} } = \mathop{v}\limits^{\rightharpoonup} _{1} + \mathop{v}\limits^{\rightharpoonup} _{2} + \mathop{v}\limits^{\rightharpoonup} _{3} + \cdots { + }\mathop{v}\limits^{\rightharpoonup} _{i} + \mathop{v}\limits^{\rightharpoonup} _{i + 1} \hfill \\ \end{gathered}$$

The *θ*_*i*_ and*θ*_*i*+*1*_ are the angles of $\mathop{V}\limits^{\rightharpoonup} _{i}$ and $\mathop{V}\limits^{\rightharpoonup} _{{i{ + }1}}$;*θ*_*i*_,*θ*_*i*+*1*_ and $\Delta \theta$ can be defined as follows:

5 $$\begin{gathered} \theta_{i} = \arctan (Y_{i} /X_{i} ) \hfill \\ \theta_{i + 1} = \arctan (Y_{i + 1} /X_{i + 1} ) \hfill \\ \Delta \theta = \theta_{i + 1} - \theta_{i} \hfill \\ \end{gathered}$$

**Figure 17:**
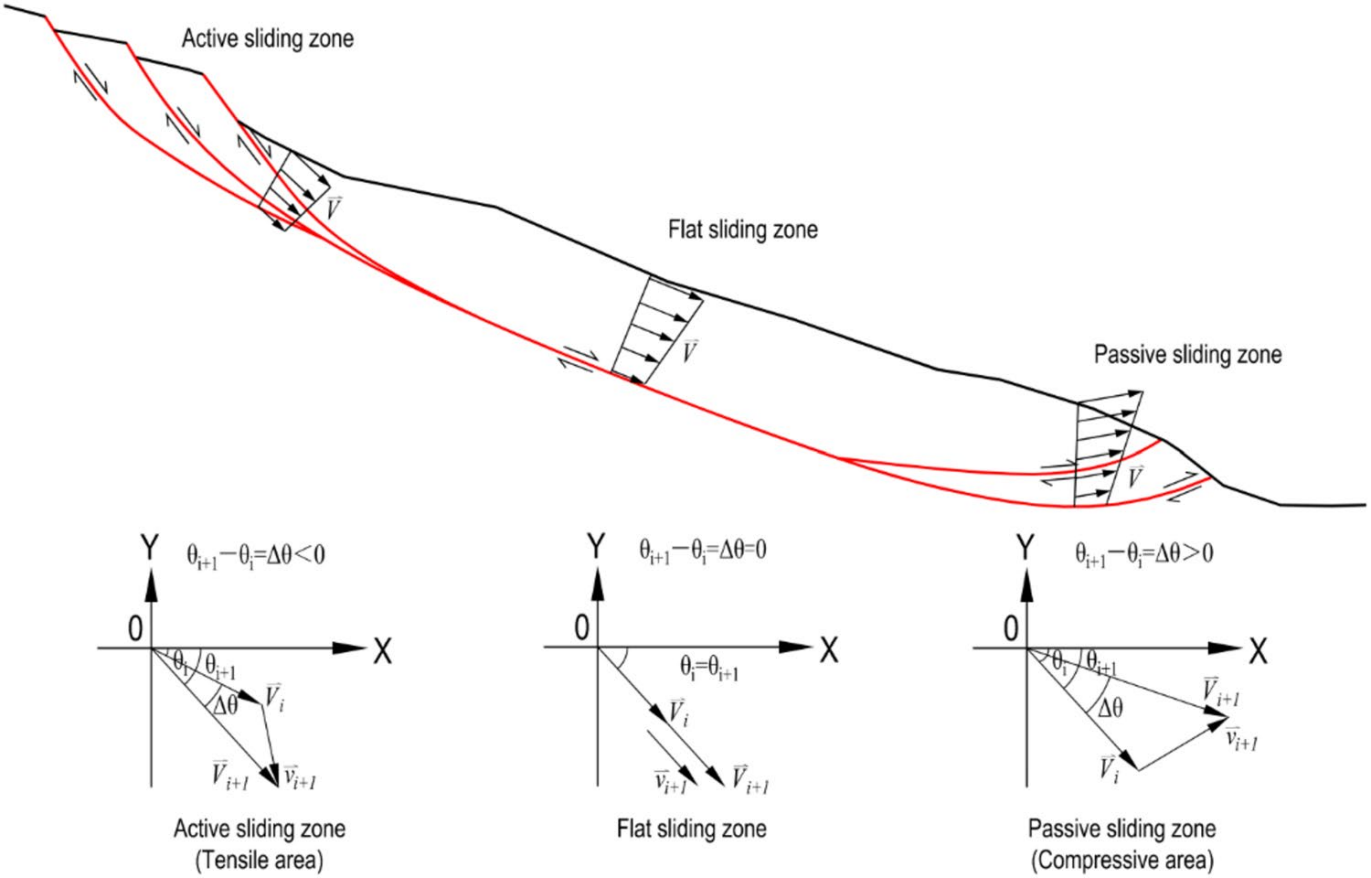
Displacement vectors angle in different sliding portions of a landslide (reproduced after He et al.^44^).

The change of this vector angle shows the stress state inside the slope. The landslide body as a whole can be subdivided into a) active sliding zones, b) passive sliding zones, and c) flat sliding zone (Fig. 17). Previous research and experience^43–47^ have shown that the active sliding zone, in which the vector angle decreases, i.e. $\Delta \theta$ < 0, controls the overall deformation of the landslide (Fig. 17). The passive sliding zone is a compressive area that resists movement, and in this zone, the vector angle increases, i.e. $\Delta \theta$ > 0 (Fig. 17). In the flat sliding zone, the vector angle does not change basically; the slope slides along the planar plane almost meet no resistance in its movement, i.e. $\Delta \theta$≈0 (Fig. 17).

**Figure 18:**
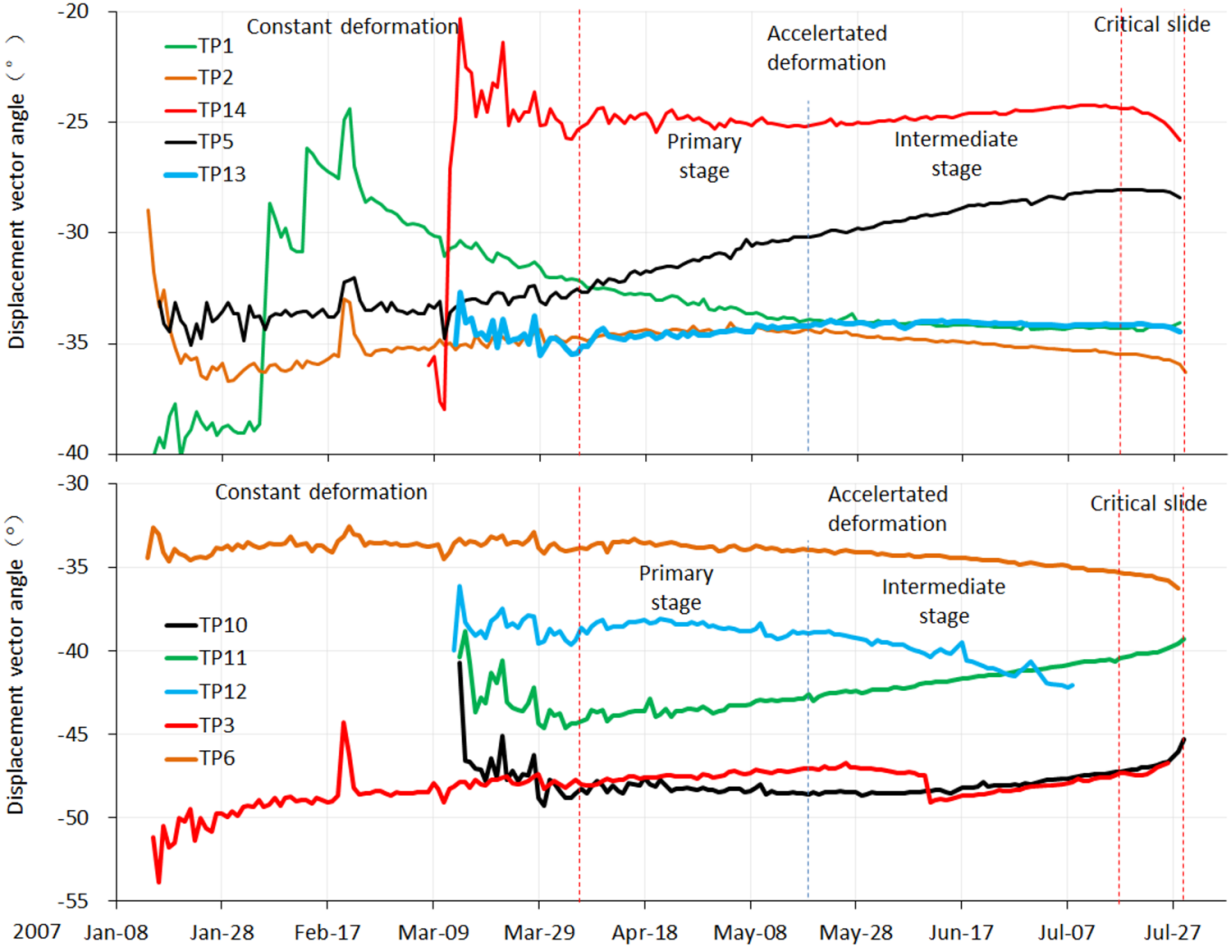
Displacement vector angle curves of the monitoring points.

This method only analyzes two-dimensional plane of the landslide, and the profile should be selected in the vertical direction of minimize deformation. By analyzing the dynamic features of surface displacement vector angles in different deformation phases, the stress state of each part of the landslide in different phases can be roughly obtained. Figure 18 shows the accumulative surface displacement vectors angle-time curve of each monitoring point, and Table 2 shows the dynamic features and stress state of each monitoring point based on data from Fig. 18, considering the measurement error, we set the valuation standard as follows: if $\Delta \theta$ < -0.5°, it is in a state of active sliding; if $\Delta \theta$ > 0.5°, it is in a state of passive sliding; if 0.5° ≥ $\Delta \theta$ ≥ -0.5°, it is in a state of flat sliding.

**Table 2:** Displacement vector dynamic features and stress state of each monitoring point in different deformation phase.

Monitoring point	Constant phase		Accelerated phase (primary)		Accelerated phase (intermediate)	
	Δθ(°)	State	Δθ(°)	State	Δθ(°)	State
TP1	8.1	Passive	− 2	Active	− 0.3	Flat
TP2	− 5.8	Active	0.3	Flat	− 1.1	Active
TP3	1.2	Passive	0.5	Flat	− 0.2	Flat
TP5	− 0.1	Flat	2.1	Passive	2.5	Passive
TP6	0.5	Flat	− 0.1	Flat	− 1.3	Active
TP10	− 7.8	Active	0	Flat	0.5	Flat
TP11	− 5.5	Active	0.4	Flat	2.6	Flat
TP12	0.4	Flat	0.5	Flat	− 3.2	Active
TP13	− 0.5	Flat	0.4	Flat	− 0.1	Flat
TP14	10.7	Passive	− 0.1	Flat	0.7	Passive

#### Constant deformation phase

**Figure 19:**
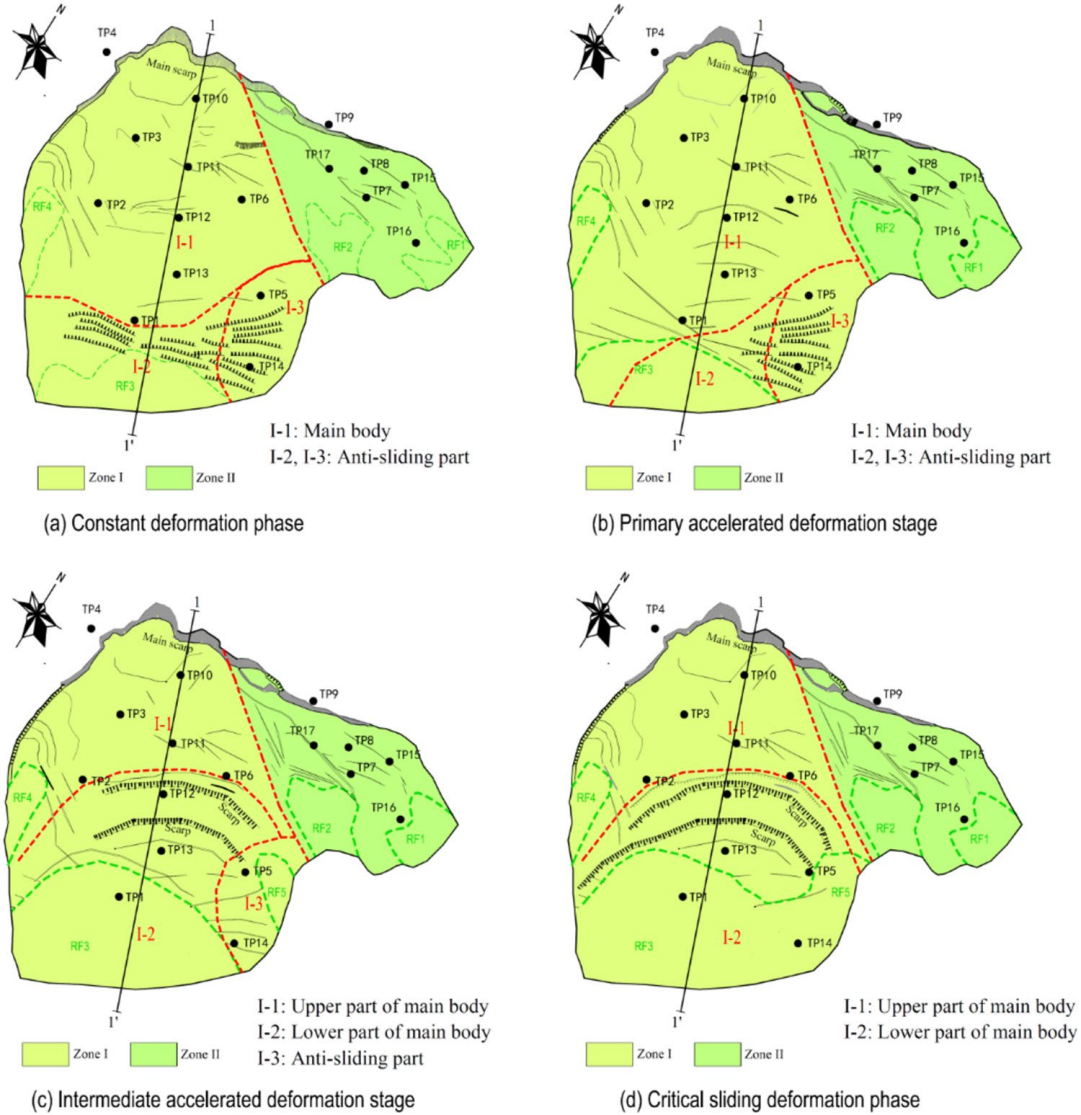
Division and deformation features of Baishi landslide in each phase: (**a**) constant deformation phase; (**b**) primary accelerated deformation stage; (**c**) Intermediate accelerated deformation stage; (**d**) critical sliding deformation phase.

**Figure 20:**
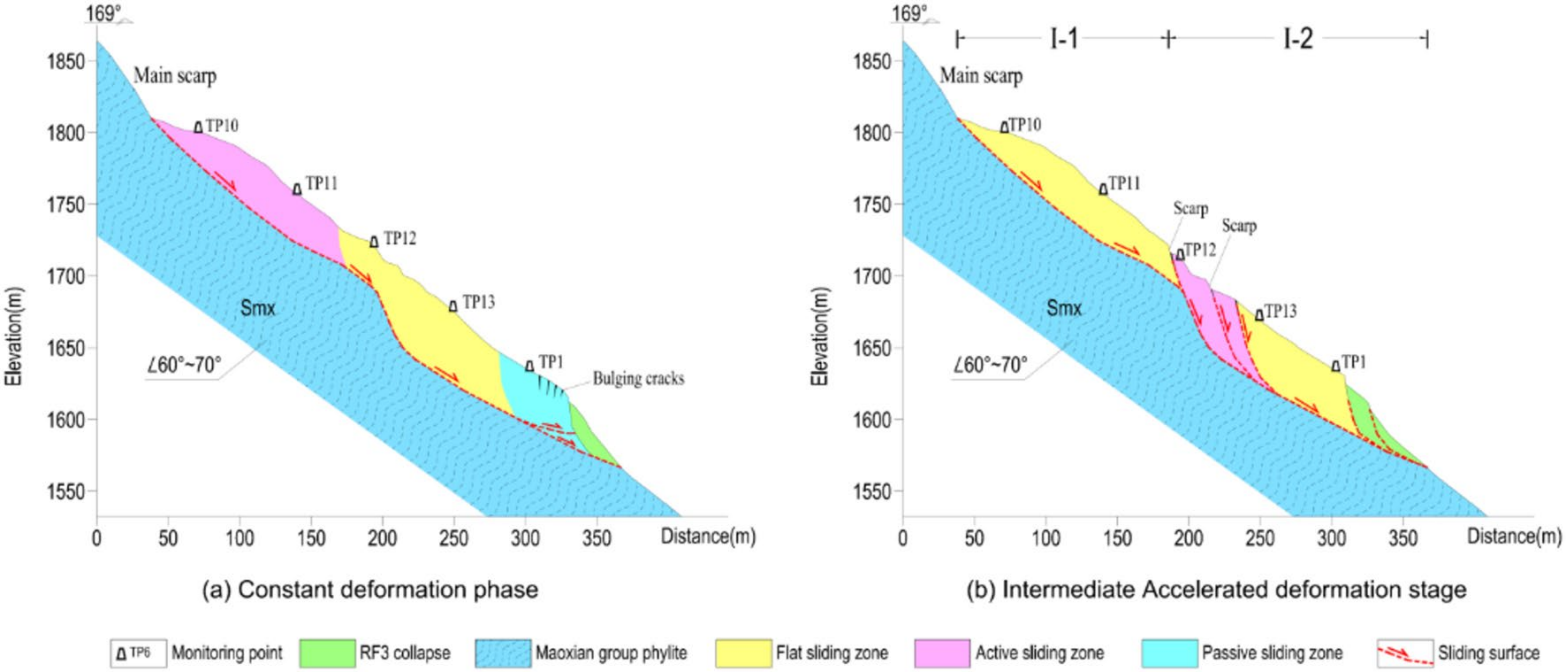
Landslide portion distribution along cross-Sect. 1–1’ during the constant velocity deformation phase: (**a**) constant deformation phase; (**b**) intermediate accelerated deformation stage.

According to the description from local villagers, the head of the landslide macroscopically deformed since the summer of 2003. The height of the main scarp was several meters at that time. Till the second half year of 2006, the height of the main scarp reached 30 m, before which, the foot of the landslide didn’t show any macroscopic deformation features. That means the accumulative displacement of the head of the landslide was much bigger than the foot before monitoring started. The TP10, TP11 (installed at the head of the landslide), and TP2 (installed in the middle part of the landslide) were in a state of active sliding, based on Fig. 18 and Table 2. However, the velocities recorded at these three monitoring points were not the highest among the ten total monitoring points (Table 1). The most probable reason is that the overall sliding body was going through the process of compaction when the monitoring started. This can be proved by analyzing the dynamic features of TP1 and TP14. The vector angle curves of these 2 monitoring points were quite different from other monitoring points in this deformation phase (Fig. 18), there was a sharp rise at the beginning and a slow decrease after that. This feature can be regarded as a strongly anti-sliding part (locked patch), where shear stress is concentrated and shows dilatancy behavior. As the potential sliding surface was in irregular geometry, which is composed of many individual joint planes, the vector angle curves were rising like steps (Fig. 18). The macroscopic deformation features show that where bulging cracks were distributed was the anti-sliding part (Fig. 19a). The anti-sliding part was divided into 2 parts, the I-2 zone (RF3 collapse) and the I-3 zone (TP5 and TP14), because of the difference in deformation features afterward. According to Fig. 18 and Table 2, in this deformation phase, the stress state of each part was similar to the ideal condition described in Fig. 17. The head part was the active sliding zone; the main body was the flat sliding zone; the foot part (toe area) was the passive sliding zone (Fig. 20).

#### The primary accelerated deformation stage

After the landslide entered the primary accelerated deformation stage, the locked patch in the I-2 zone started to be broke through, the anti-sliding part in the I-2 zone was narrowed during this deformation stage (Fig. 19b), shown by the expansion of RF3 collapse boundary, the volume of rock fall happened in RF3 increased day by day, so TP1 who near RF3 collapse was in a state of active sliding (Table 2). Meanwhile the macroscopic deformation of I-3 zone (Fig. 19b) almost remained the same. Upon comparing Figs. 19a and b, the expansion of the I-2 zone happened on the right side, the left side where contact with I-3 zone basically did not change. These two features show the strongest anti-sliding effect in the I-3 zone of the landslide. So TP5 installed in the I-3 zone was in a state of passive sliding (Table 2). Other monitoring points were in a state of flat sliding (Table 2).

#### Intermediate accelerated deformation stage

**Figure 21:**
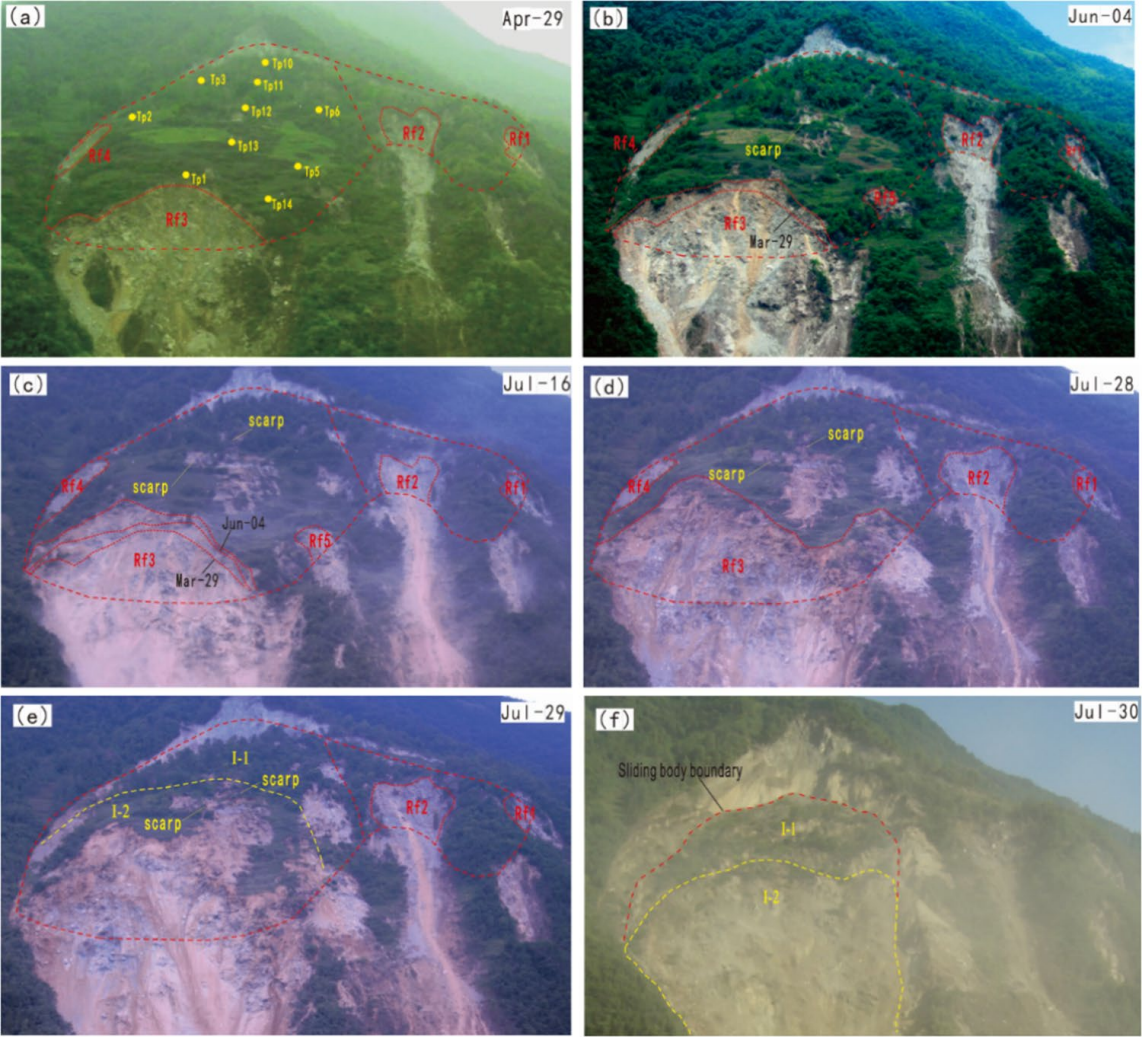
Full view of Baishi landslide in different deformation phases: (**a**) Mar 29, 2007, in constant deformation phase; (**b**) Jun 4, 2007, in intermediate accelerated deformation stage; (**c**) Jul 16, 2007, right before the entry of critical sliding deformation phase; (**d**) overall slide body started to collapse on Jul 28, 2007; (**e**) overall slide body was collapsing on Jul 29, 2007; (**f**) overall slide body was collapsing on Jul 30, 2007.

Comparing to the boundary variation of RF3 in last deformation stage (Fig. 21a), RF3 expanded more rapidly after the entry of this deformation stage (Figs. 21b, and c). After 3 large-scale rockfall events occurred in RF3, the locked patch structure was completely destroyed, the anti-sliding part in that area (I-2 zone in Fig. 19b) lost its effect. The whole sliding surface was almost developed, except the I-3 zone still played an anti-sliding role (Fig. 19c), which can be proved by TP5, TP14 was in a passive state (Table 2). But the occurrence of RF5 collapse at the beginning of this stage showed the locked patch in I-3 zone started to be broke through. After the sliding surface was almost developed, the deformation features and mechanism were controlled by the geometry of the sliding surface. After the overall failure of the landslide, the exposed sliding surface was measured. According to the measured data, on Sect. 1–1’, from the main scarp to TP11, the sliding surface was almost planar; the dip angle of sliding surface between TP11 and TP12 was decreased; at the place of TP12, the sliding surface became steep, then became gentle at the TP13; from TP13 to the shear outlet, the sliding surface became almost a planar (Fig. 20). The special shape of sliding surface leads to the retrogressive deformation features during this deformation stage. Two fissures near TP12 and TP11 expanded rapidly and finally formed two scarps, which can be observed in Figs. 21b and c. The main body was divided into two parts by scarps, the upper part I-1 zone and the lower part I-2 zone (Fig. 19c). Three rock fall events in RF3 caused the collapse and unloading of the lower part, resulting in the formation of the lower part. The activity of the middle part of the landslide was enhanced, illustrated by TP2, TP6, and TP12 were in a state of active sliding (Table 2). The flat sliding zone was defined as moving with no resistance, so where the sliding surface was totally formed, the sliding body was in a state of flat sliding, including TP1, TP3, TP10, TP13 (Table 2, Fig. 20).

#### Critical sliding deformation phase

In the critical sliding deformation phase, RF5 collapsed and deformed rapidly, when the locked patch of there was totally broken through, the boundaries of RF3 and RF5 became one (Fig. 19d, Fig. 21d). The movement of the landslide was primarily influenced by unloading in the toe area of the landslide. The shear outlet was totally exposed, and the overall failure began. From Figs. 21e and f, the scarps dividing I-1 zone and I-2 zone can be observed during the overall sliding process.

## Conclusions

Based on the deformation phases obtained from the improved tangential angle criterion, and combining these with the macroscopic deformation features, we could conclude that the failure mechanism of the Baishi landslide didn’t deform in one fixed mode. During the constant velocity deformation phase, the whole landslide body showed characteristics of advancing mode, where the upper part was the active sliding zone which was pushing the middle and the front part. During the accelerated deformation phase the landslide showed some characteristics of retrogressive mode, analysis of the monitoring data shows that the middle part was then the active sliding zone and the most dynamic part of the landslide. The upper part acted as a passive sliding zone. Due to the differences in velocities, the main body was separated into two parts.

The failure mechanism analysis indicates that the toe area of the landslide plays a key role in controlling the stability of the Baishi landslide during the critical slide phase. Therefore, the monitoring data of this zone were significantly important for the prediction of the moment of failure of the whole landslide. Identifying the signs of the stability change of this key part in due time by a careful analysis of the monitoring data turned out to be the right approach for a realistic early warning. Correct recognition of the evolution phases of a landslide is the basis for accurate landslide early warning, by identification of the accelerated deformation phase as the start of a landslide failure. During the constant velocity deformation phase the landslide will not fail entirely under normal circumstances, no matter how high the deformation velocity and displacement are, and how severe the macroscopic deformation is.

## Data Availability

The datasets used and/or analysed during the current study available from the corresponding author on reasonable request.
