# Author Correction: The failure mechanism of the Baishi landslide in Beichuan County, Sichuan, China

**DOI:** 10.1038/s41598-025-90716-7

**Published:** 2025-02-18

**Authors:** Ran Tang, Suichuan Ren, Juntao Li, Peng Feng, Huajin Li, Ren Deng, Daxin Li, Kiyonobu Kasama

**Affiliations:** 1 School of Architecture and Civil Engineering, Chengdu University, Chengdu, Sichuan China; 2 Key Laboratory of Hydraulic and Waterway Engineering of the Ministry of Education, Chongqing Jiaotong University, Chongqing, China; 3 Sichuan Engineering Research Center for Mechanical Properties and Engineering Technology of Unsaturated Soils (Chengdu University), Chengdu, Sichuan China; 4 Sichuan Institute of Geological Engineering Investigation Group Co.Itd, Chengdu, Sichuan China; 5 Department of Civil Engineering, Kyushu University, Fukuoka, Japan

Correction to: *Scientific Reports* 10.1038/s41598-024-67402-1, published online 30 July 2024

The original version of this Article contained an error in the spelling of the author Suichuan Ren, which was incorrectly given as Suichun Ren.

The original Article has been corrected.

